# Editorial: Women in avian physiology 2025

**DOI:** 10.3389/fphys.2026.1912029

**Published:** 2026-07-28

**Authors:** Sandra G. Velleman, Krystyna Pierzchala-Koziec

**Affiliations:** 1 Department of Animal Sciences, The Ohio State University, Wooster, OH, United States; 2 University of Agriculture in Krakow, Krakow, Poland

**Keywords:** avian physiology, eggshell, extracellular matrix, immunity, microbiome, skeleton, stress, virology

## Introduction

The Women in Avian Physiology: 2025 is the third research topic in the Frontiers Avian Physiology section focused on women. The objective of this series is to help promote avian physiology research being led by women who are especially early in their careers. Submissions are welcomed from all areas of Avian Physiology. In the 2025 topic, 9 papers were accepted covering the areas of Immunity and the Microbiome, Satellite Cells and Extracellular Matrix, Skeletal Integrity and Eggshell Production, Stress, and Avian Influenza.

## Immunity and the microbiome

Dasireddy et al. studied the use of symbiotics to regulate the cecal microbiota in chickens through enhancing the growth of beneficial bacteria to limit the negative effects of mycotoxins found in feed and to improve the immune response. Chronic ingestion of mycotoxins impacts intestinal function. They found that synbiotic supplementation on day 35 in chickens increased mucosal immunity and modified microbial composition and fermentation profiles. This is a first step in the research to develop *in vivo* microbial detoxification strategies to improve gut health.

Nabthonglang et al. and colleagues studied the effect of bardoxolone methyl on the lower reproductive tract microbiome in turkey breeder hens. In turkeys, fertility declines with age due to reduced sperm storage in the uterovaginal junction from oxidative stress, inflammation and aging. Bardoxolone methyl reduces both inflammation and oxidative stress. Their results showed that bardoxolone methyl treatment enhanced energy metabolism, nucleotide biosynthesis, and stress resilience without altering microbial diversity but may fine-tune mucosal microbial communities in the uterovaginal junction.

Many wild bird populations are in decline from environmental changes. Krama et al. researched the blood microbiome in wintering greenfinches before and during an outbreak of trichomonosis. Prior to the outbreak no bacterial contamination was found in the blood. Interestingly greenfinches with irregular food access had lower bacterial contamination in their blood compared to birds with permanent food access. Thus, feeding accessibility affects microbial contamination, and may play a role in the physiological resilience in wild birds.

## Satellite cells and extracellular matrix

A review article by Yimiletey et al. discussed how intense genetic selection in commercial poultry has increased growth rate, breast muscle mass, and production efficiency. The satellite cell is responsible for all posthatch muscle growth. With genetic selection the satellite cell population has changed in its cellular properties of proliferation and differentiation as well as molecular profiles and heterogeneity. These changes in the satellite cell population in commercial birds may significantly contribute to the development of breast muscle myopathies including wooden breast. Further defining satellite cell biology will benefit commercial poultry production systems.

Wooden breast in broilers is characterized by the fibrosis of the breast muscle. The transmembrane proteoglycan, syndecan-4, is a key regulator of satellite cell and fibroblast proliferation. Pejšková et al. have found through the over expression of syndecan-4 in fibroblasts that it may be a modulator of the fibrotic response in chickens.

## Skeletal integrity and eggshell production

Keel bone fractures and deviations are an animal welfare issue in laying hens. Fractures of the keel bone lead to reduced mobility and egg laying. Hildebrand et al. compared keel bone traits and eggshell production in a hybrid layer line and two non-egg laying chicken genotypes. They found that earlier onset of egg laying in the hybrid layer line did not correspond with earlier maturation of the keel bone. The delayed maturation of the keel bone in the hybrid layer line in relation to eggshell production could increase susceptibility to fractures of the keel bone.

Leskovec et al. reported on the effects of n-3 polyunsaturated fatty acids (PUFA) and heat stress on the morphology and geometry of the tibiotarsal growth plate in Ross 308 broilers. Interestingly, high PUFA dietary supplementation induced morphometric changes in the growth plate that were similar to tibial dyschondroplasia. Heat stress reduced the crude ash content and biomechanics of the tibiotarsal growth plate. Adding antioxidant supplementation to the feed negated the effects of high PUFA levels and heat stress. Taken together, these findings may lead to the development of nutritional strategies to improve broiler bone health.

## Stress

Pierzchala-Koziec et al. showed that not only does stress induce increased plasma concentrations of Met-enkephalin in chickens but also peripheral administered Met-enkephalin depressed the hypothalamo-pituitary-adrenal axis (HPA) axis activity during restraint stress. Thus, it is proposed that endogenous Met-enkephalin modulates the activity of the HPA axis.

## Avian influenza

Infection with avian influenza virus can negatively impact reproduction in turkey breeder hens. Kosonsiriluk et al. reported on the early transcriptomic response to low-pathogenic strain avian influenza infection in the vagina and uterovaginal junction. In the vagina, 701 genes were differentially expressed in symptomatic vs. presymptomatic birds. Pathways affected in symptomatic turkey hens included those associated with protein synthesis, metabolism energy production, and vascular development. In the uterovaginal junction pathways involved in cellular maintenance and metabolism were affected.

